# Safety and efficacy of percutaneous patent ductus arteriosus closure: a multicenter Egyptian experience

**DOI:** 10.1186/s43044-022-00251-3

**Published:** 2022-03-04

**Authors:** Amira Nour, Yasmeen Abdelrazik, Safaa Huessin, Heba Kamel

**Affiliations:** 1 Congenital and Structural Heart Disease Unit, Cardiology Department, Ain Shams University Hospital, P.O. 11835, Abbassya, Nargess 3, Fifth Settlement, Cairo, Egypt; 2 Pediatric Department, Sohag University, Sohâg, Egypt

**Keywords:** Patent ductus arteriosus, Transcatheter closure, Complications

## Abstract

**Background:**

Transcatheter closure of patent ductus arteriosus (PDA) has gained acceptance over the last two decades, replacing the surgery in more than 90% of the cases, so the safety and efficacy of transcather closure of PDA have been evaluated by studying different experiences from different centers in developing countries. The aim is to report our experience with PDA transcather closure, with focus on the adverse events and complications faced during the procedure.

**Results:**

Outcome data on PDA transcatheter closure were collected from two different tertiary centers in a multicenter registry. During the period from June 2017 till January 2021, 308 PDA closure were recorded, using device in 197 (64%) and coils in 111 (36%) patients, most of the patients were in pediatric age group from 6 months to 6 years and only 10 patients (3.2%) were adults. Most patients had isolated PDA of 92%, and 9 (2.9%) patients had residual PDAs either post-surgical or transcatheter closure. Median minimum PDA diameter was 2.8 mm (range 1–7.6 mm; IQR 1.8–3.8 mm). The procedure was successful in 293 patients (95%). Complications occurred in 15/308 patients (5%), and only 6 (2%) of them were major complications, but none was life threating. Frequent complications were device embolization (2%), hemolysis (1%), arrhythmia (1%). Younger age, low body weight and longer procedure time were associated with a high complication rate (*p* < 0.005). Device-related complications were more common than coil-related complications (2.5% versus 0.5%).

**Conclusions:**

Although transcatheter closure of PDA is considered to be effective procedure with low complications rate, however, complications should be anticipated and managed properly.

## Background

Patent ductus arteriosus (PDA) accounts for 5–10% of all congenital heart diseases and is estimated to occur approximately 1 in 2000 live births [1, 2]. The natural history and clinical picture of PDA vary widely and are largely dependent on the size of PDA, degree of shunting and pulmonary vascular resistance (PVR). Patients with small-sized PDA are usually asymptomatic, and patients with moderate sized to large PDA may complain of symptoms of volume overload, left-sided heart failure, failure to thrive and recurrent chest infection [3]. If a patient with large PDA was not corrected and left without protective measures, persistent pulmonary hypertension (PH) and consequent Eisenmenger syndrome develop [4]. In rare cases complications as ductus aneurysm, ductus calcification and endarteritis may occur, so transcather PDA closure is often sought preferably at an earlier age [5].

After Portsmann described the first successful transcatheter closure of PDA in 1971, the procedure became widespread in the 1980s [6]. Transcatheter closure of PDA has become the standard of care for most cases, reserving only the surgical options for very few cases. With large technical advances in the devices used for pediatric cardiac interventions, even large PDAs are now amenable for transcatheter closure [2]. The most two transcatheter techniques used are coil embolization or device closure, with large variety of devices now being commonly used as Amplatzer duct Occluder (ADO I, II), Hyperion, lifetech and occlutech with high success rate and less incidence of complications [7]; however, reports from developing countries are lacking. So, we find it important to document our multicenter study regarding success rate, rate of complications, level of experience and level of care offered during and after the procedure.

## Methods

This is a prospective non-randomized study done in X university hospitals in conjunction with XX university hospital, and both are tertiary referral centers with well-established congenital and structural heart disease service.

This is a prospective study for evaluation of the patients who underwent PDA closure, reporting our experience, short- and long-term outcomes and incidence of complications.

From January 2017 till January 2020, all patients with PDA after signing an informed consent (both pediatrics and adults), and fulfilling inclusion criteria, audible hemodynamically significant PDA (either evidence of left ventricle (LV) volume overload or QP/QS > 1.5) were included.

While patients with irreversible severe PH (PVR > 5 WU) after balloon occlusion test, or in those where the surgical option would be preferred (as preterm infants, PDA with complex anatomy, or in whom the device will be too large in relation to their BSA).

A custom-made sheet was made to include all relevant demographic data which included age at first presentation, age on the day of procedure, body weight, height, transthoracic echocardiographic (TTE) data (size by TTE, LV end diastolic volume index and end systolic volume index and ejection fraction), associated lesions, procedural data as (fluoroscopy time, procedural time, size of PDA by angiography, QP/QS, mean pulmonary artery pressure (PAP)).

In our study, patients with PDA 2 mm or less underwent coil implantation, while PDA more than 2 mm underwent device implantation instead. Success of implantation was defined as the device being properly placed and deployed without malposition or embolization in the catheterization laboratory or significant residual defect. Immediate and short-term complications were recorded further classifying them into major and minor complications.

All patient underwent detailed history taking, physical examination, chest X-ray (CXR), electrocardiogram (ECG), detailed TTE then scheduled for transcatheter closure, after written informed consent the interventional procedure with different devices or coils was carried out under general anesthesia. All patients received prophylactic antibiotic therapy prior to catheterization. After the percutaneous puncture of the femoral vein and artery unfractionated heparin (100 unit/kg) was given, then all hemodynamic variables were evaluated. The shape and size of the PDA were then assessed via an aortogram, from the right anterior oblique and lateral projections. The ductal shape was classified in accordance with the classifications established by Krichenko et al. [8]. Depending on the basis of ductal morphology and size, transcatheter closure was conducted. The devices used were 1–2 mm larger than narrowest diameter of the pulmonary end of the PDA and were implanted via a venous approach, while using either cook coil or Nit-Occlud coil the largest device loop diameter was equal to the aortic ampulla and was 3–4 mm larger than the narrowest duct diameter and was deployed via the venous approach or arterial approach.

TTE evaluations and chest radiography were conducted on all patients prior to discharge, and at 1, 3, 6, 12 months after the procedure. The outcomes of interest for the study included acute complications at the time of the procedure, and during follow-up at 1 month, 3 months, 6 months and one year after discharge.

### Statistical analysis

Collected data were coded, tabulated and statistically analyzed using IBM SPSS statistics (Statistical Package for Social Sciences) software version 22.0, IBM Corp., Chicago, USA, 2013. Descriptive statistics were done for quantitative data as minimum and maximum of the range as well as mean ± SD (standard deviation) for quantitative normally distributed data, while it was done for qualitative data as number and percentage. Correlations were done with the level of significance applied at a *p* value < 0.050 which is significant, and otherwise, it is non-significant.

## Results

**Fig. 1:**
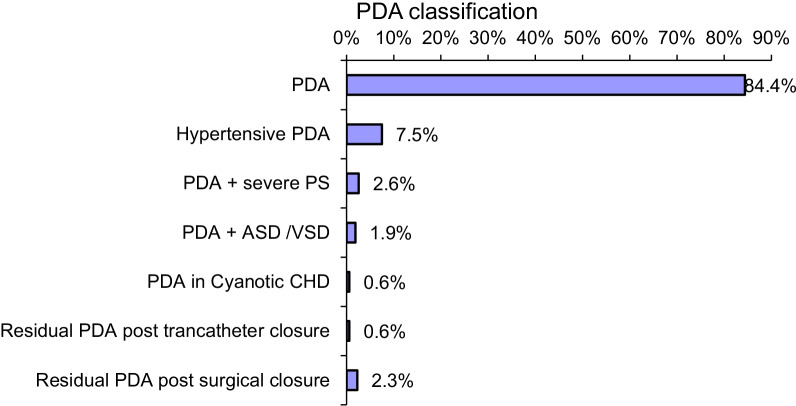
Classifications of patent ductus arteriosus. *ASD* atrial septal defect, *CHD* congenital heart disease, *PDA* patent ductus arteriosus, *PS* pulmonary stenosis, *VSD* ventricular septal defect

This study evaluated 308 patients, and transcatheter closure was conducted successfully in 302 patients (98%) using different devices and coils. The median age and weight for the patients were 2.7 years (range from 3 months to 20 years) and 11.8 kgs (range from 5 to 90 kgs), respectively. We recorded 260 cases (84.4%) of isolated PDA, 23 patients (7.5%) with hypertensive PDAs (who underwent balloon occlusion test), 14 patients (10.1%) with PDA and other congenital heart diseases, 2 patients (0.6%) with PDA post-Glenn in patients with congenital cyanotic heart diseases. Nine patients (2.9%) had residual PDAs either post-surgical or transcatheter closure (Fig. 1).

The mean procedural time was 53 ± 6 min, mean Fluoro time was 6.8 ± 0.86 min, the median size for PDA (narrowest PDA diameter) by angiography was 2.86 ± 0.96 min (range 1.5–7.6 mm), the median of mean PAP was 15.5 mmHg (range 10–55 mmHg), and the median QP/Qs was 2.15 ± 0.8. According to the classifications established by Krichenko et al., the PDA assumed a conical shape (type A) in 250 patients (81%), a tubular shape (type c) in 48 patients (15.5%), a window-like (type B) morphology in 8 patients (2.5%) and complex (type D) morphology in 2 patients (0.6%).

Percutaneous PDA closure using device was done in 208 patients (67.5%), and using coil was done in 100 patients (32.5%). The most common device used was ADO I in 132 patients (42.8%) followed by Hyperion in (8.1%) of patients, then Occlutech in 7.8% of the patients, then lifetech in 3.8%. Muscular ventricular septal defect (VSD) was used in 10 patients (3.2%) with hypertensive PDA, and ADO II was used in 4 patients (1.29%), while cook coil was used in 90 patients (29.2%) and PFM coil in 10 (3.2%), and the median device size was 6 mm at pulmonary end (range from 4 to 12 mm) (Table 1).

**Table 1:** Demographic data

Age	Median = 2.7 years (range 3 months–20 years)
Gender	Males, *n* = 113 (36.7%), females, *n* = 195 (63.3%)
Weight	Median = 11.8 kgs (range 5–90 kgs)
Size of PDA by angiography	Median = 2.86 ± 0.96 mm (range 1.5–7.6 mm)
Mean PAP	Median 15.5 mmHg (range 10–55 mmHg)
QP: Qs	Median 2.15 ± 0.8 (1–3.5)
Procedural time	Mean 53 ± 6.8 min (range 42–70 min)
Fluoroscopy time	Mean 6.8 ± 0.86 min (range 6–9 min)
Type of intervention	Device closure, *n* = 208 (67.5%), coil closure *n* = 100 (32.5%)
Type of PDA	Type A *n* = 250 (81%), type C, *n* = 48 (15.5%), type B, *n* = 8 (2.5%), type D, *n* = 2 (0.6%)
Device type	ADO I *n* = 132 (42.8%), Hyperion *n* = 25 (8.1%), Occlutech *n* = 24 (7.8%) Lifetech *n* = 12 (3.8%). Amplatzer Muscular VSD, *n* = 10 (3.2%), ADO II, *n* = 4 (1.29%)
Device size	Median 6 mm (range from 6 to 12 mm)
Complications	*n* = 15 (5%)

*PDA* patent ductus arteriosus, *PAP* pulmonary artery pressure, *VSD* ventricular septal defect

### Major complications

The overall complication rate was 5% (15 out of 308 patients), major complications were reported in 6 patients (2%), 4 of them had device/coil embolization, and 3 patients had device embolization. Case 1 was a 9-month-old female patient, with type C PDA measuring 4.3 mm, and ADOI 6/8 was deployed across; however, immediately after the release it embolized into main pulmonary artery, the device was snared, and then, patient was sent for surgical closure. Case 2 was a 11-month-old boy with type A PDA, pulmonary end was 2 mm, an ADO I 5/4 was used with no residual shunt after device deployment; however, immediately after release the device embolized in the descending aorta with failure of percutaneous retrieval and then the device was extracted surgically followed by PDA surgical closure. Case 3 was a 10-month-old female patient, had small-sized type C PDA measuring 1.5 mm, closure using ADO I 5/4 mm was attempted; however, the device immediately embolized into right pulmonary artery and snaring failed percutaneously and patient was sent for surgery. The reason for embolization in Cases 1 and 2 is mostly due to under-sizing; however, in Case 3 it is mostly due to abnormal morphology of the duct as the device after it has squeezed. While Case 4 was 4 years old, PDA pulmonary end size was 2.5 mm. We decided to close it using PFM coil 7 × 6 mm, however , unfortunately during device release. It was entrapped in the pulmonary valve leaflets, and then, it jumped into the right ventricle and again become entrapped with the tricuspid valve (TV) leaflets and could not be snared, and the patient was sent for surgery immediately with successful device extraction and repaired one of the TV chordae with mild tricuspid regurgitation and PDA surgical closure.

Two patients underwent significant hemolysis requiring blood transfusion after PFM coil implantation. Case 1 was a 9-year-old female patient with type C PDA measuring 3.5 mm, and we decided to close it with PFM coil 9 × 6 mm. Post-coil release injection showed minimal residual flow across and two weeks later in the follow-up TTE showed significant residual flow across His hemoglobin dropped from 12 to 8 g/dl and increased in the indirect bilirubin and retics, so the patient admitted to the cath. laboratory again, and there was a significant residual with QP/Qs > 2, so another cook coil 5 × 5 was deployed with complete closure of the PDA. Case 2 was a 2-year-old boy with PDA measuring 3.5 mm at the narrowest diameter who underwent PDA device closure using ADO I 8/6 with minimal residual flow across, and in the follow-up echo, there was a significant residual flow. The patient was pale his hemoglobin drop to 8 g/dl, patient was admitted received blood transfusion then re-entered the cath. laboratory closing the residual defect with a cook coil 5 × 5 (Table 2).

**Table 2:** Incidence and types of complications

Category	Complication	N	Percentage	Total (%)
Major complications	I. Embolization	4	1.3	2
	Device embolization	3		
	Coil embolization	1		
	II. Major vascular complication (hemolysis requiring blood transfusion)	2	0.6	
Minor complications	I. Minor Vascular complications	3	1	3
	II. Benign arrhythmia (SVT and atrial tachycardia)	3	1	
	III. Device encroachment on descending aorta or LPA	3	1	
Total		15	5	5

*LPA* left pulmonary artery, *SVT* supraventricular tachycardia

### Minor complications

#### Minor vascular complications

Minor complications were recorded in 9 patients (3%), and these complications included minor vascular complications in 3 patients (1%). Two of them developed small hematoma at the puncture site; however, there was no significant blood loss, hemoglobin dropped only by 1–2 g/dl, and the hematoma was managed conservatively until it regressed after 48 h. One patient (6 months) developed arteriovenous (AV) fistula after PDA closure as evidenced by bruit on auscultation, and connection between femoral artery and vein by ultrasonography; however, there was no limb swelling or discrepancy in size between the two limbs, and no evidence of ischemic changes patient was being followed up for two months with spontaneous resolution of the chronic AV fistula without the need for surgical intervention.

#### Benign arrhythmia

Arrhythmia was recorded in 3 patients (1%), two of them were 2 years and 4 years, respectively, who developed supraventricular tachycardia (SVT) immediately after the procedure (mostly due to anesthetic drugs), and vagal maneuvers were tried, but with no effect, patients received adenosine, where the normal sinus rhythm was restored with no recurrence of arrhythmia again. A 9-month-old boy patient with large PDA measuring 3.5 mm, dilated LV and dilated left atrium after PDA device closure developed atrial tachycardia which was reverted using also adenosine giving twice; however, patient has recurrence of arrhythmia later on which is mostly intrinsic and not iatrogenic due to device implantation, and then, the patient was referred for the electrophysiology team for further management.

#### Device encroachment on the descending aorta or peripheral pulmonary tree

**Fig. 2:**
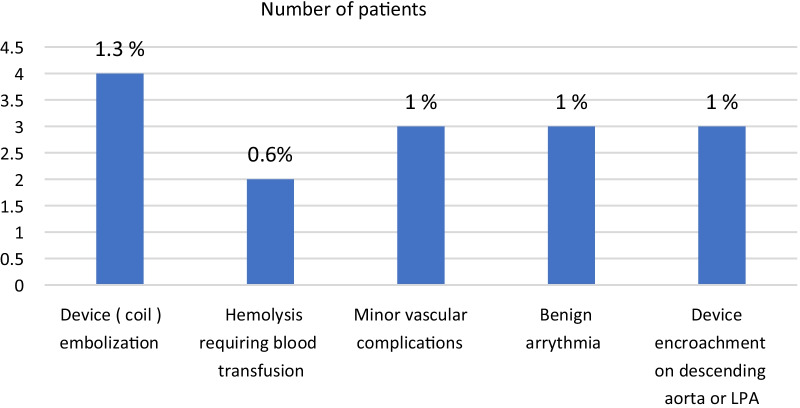
Patent ductus arteriosus complications. *LPA* left pulmonary artery

We had only one patient (10 months old) with large hypertensive PDA which was closed by Occlutech 10/8 mm device, the device was minimally protruding into the descending aorta with pressure drop across invasively of 4 mmHg and pressure drop across by Doppler of 12 mmHg, and the patient has been followed up for 1 year with no increase in the Doppler pressure gradient across aortic ismuth. Another case of a 6-year-old girl with large hypertensive PDA was reversible after balloon occlusion test, and a muscular VSD hyperion device 12/10 was deployed with minimal encroachment on the left pulmonary artery (LPA) with non-significant Doppler pressure gradient across LPA invasively; however, 2 months later in the follow-up there was mild origin LPA stenosis with Doppler PPG across of 14 mmHg. There was another case with large PDA closed by muscular VSD Amplatzer device which ended up with mild LPA origin stenosis with Doppler gradient across of 16 mmHg (Table 2, Fig. 2).

On comparing non-complicated to complicated group, we find that patients with hypertensive PDA (with elevated mean PAP) are at higher risk for complication (*p* value = 0.010), and other risk factors including low body weight (*p* = 0.023), procedural time (*p* = 0.031), fluoroscopy time (*p* = 0.021), device size (*p* = 0.042) were statistically significant in the complicated group; however, there was no statistical significant difference between the use of either coil or device method for closure between the two groups.

## Discussion

Since PDA transcatheter closure is among the safest interventional cardiac procedure and is now considered the method of choice for PDA closure in infants above 5 kgs [9], a variety of devices have been approved for safe and effective PDA closure [10]. In our study, we achieved 95% success rate with only 5% rate of adverse events, major complications accounted for 2% (6 patients), and the rest 3% (9 patients) had minor complications in patients with minimum size of PDA 1.5 mm and maximum up to 7 mm. Device embolization was reported in 4 patients, and 2 patients had significant hemolysis requiring blood transfusion, 3 patients underwent minor vascular complications in the form of hematoma and arterial venous fistula, 3 patients developed benign arrhythmia, and 3 patients had minimal device encroachment either on LPA or descending aorta.

In comparison with G.Y. Jang and his colleagues, who reported an overall complication rate of 8.5% [11], 4 major complications (3.4%) and 6 minor complications (5.1%), reported complications were in the form of device embolization requiring surgical interventions, significant hemolysis requiring transfusion, infective endocarditis, benign arrhythmia, mild device encroachment on LPA or descending aorta.

A higher complication rate was reported by El-Said et al. reaching up to 9%, 2.2% (24 patients) of them were defined as high severity events in the form of device embolization (11 patients) or malposition (13 patients), while the rest of complications were minor in the form of minor bleeding, anesthesia or airway-related complications, arrhythmia, allergic reactions and medication errors [12].

A Shigini and his co-workers in their multicenter study in Iran reported no major complications as device embolization, and they only reported minor complications in the form of frequent benign arrhythmia and less commonly hemorrhage [4].

Device embolization is a rare complication and was reported in our early experience with PDA device closure mostly due to under-sizing or abnormal morphology of the duct, and the rate of device embolization was variable in the literature with max rate up to 16% in the early experience; however, with more experience, availability of different devices, understanding the different morphology of the duct, decreased to zero % [13]. El Said et al. reported that the incidence of embolization was more commonly seen in the coil (5%) rather than device (1%) [12]. In contrast to our study, we had more device embolization than coil; however, it was not statistically different. However, many registries reported that coil embolization is more commonly seen with large PDAs, so device would be a better option for PDAs more than 2.5 mm, and device embolization was also commonly reported due to under-sizing [14], and however, this rate of coil or device embolization decreased due to improved coil and catheter technology available for coil implantation, including detachable coils and the continuous efforts of investigators to improve the efficacy and safety of coil or device placement [15].

Significant hemolysis after device deployment is primarily related to residual shunts and occurs only rarely. This is believed to result from mechanical injury to the red blood cells, and the severe hemolysis appeared to have been associated with coil deployment and residual shunts; especially if large coils deployed in large-sized PDA, there is increased incidence of residual shunting and consequently hemolysis [16]. The usual management of hemolysis involves the closure of the residual shunt, either surgically or via the deployment of the appropriate device; in our study the two cases were managed percutaneously.

Transcatheter closure of large hypertensive PDA after balloon occlusion test was successfully conducted in 23 patients (7.5%); however, this increased the rate of minor complications as we commonly use larger double disk devices for those patients. Also using a large device in infants weighing less than 10 kgs is quite problematic. Our main concern regarding morbidity in relation to this device would be significant obstruction of the descending aorta and LPA. Therefore, in case we use large PDA devices in relation to the patient’s body surface area. Aortic pressure gradient should be constantly measuring before device release or if it encroaches on LPA, also pressure gradient across should be recorded across, and in certain cases, we can use a balloon dilatation technique to the LPA if there was mild LPA stenosis before device release [17]. In our study, we had only three cases with mild encroachment on the descending aorta or LPA which was managed conservatively with no pressure gradient across. Congruent to our results, G.Y. Jang reported 5% incidence rate of mild narrowing of LPA or descending aorta with no need for surgical interventions [11].

The overall incidence of complication rate did not differ significantly between coil and device implantation; in our study, despite that it was a little bit higher in the coil group (3% vs 2% *p* > 0.05). El Said et al. reported in contrast to our study that coil-related complication rate was higher than device-related complication rate (10% vs 2% *p* < 0.001) [12], while A Shigini and his colleagues reported that the complication rate did not differ significantly between the two techniques (*p* > 0.05) [4].

On multivariate analysis for the risk factors, we find that low body weight, elevated pulmonary artery pressure (hypertensive PDA), procedural time, fluoroscopy time and device size are single independent risk factors for higher complication rate. Our results were comparative to El Saed et al., and she reported that higher event rates were more likely to occur with younger patients, low body weight and PH [12].

## Limitations

This a retrospective single-treatment arm study, not comparing the catheter-based outcomes and complications to that of the surgical therapy.

## Conclusions

PDA transcatheter closure is safe and effective procedure; however, to prevent complication rate case-by-case approach is needed considering patient’s condition, ductal anatomy, operator’s experience, appropriate device selection and availability of surgical backup.

## Data Availability

The datasets used and analyzed during the current study are available from the corresponding author on reasonable request.

## Abbreviations

AV: Arteriovenous
BSA: Body surface area
CXR: Chest X-ray
ECG: Electrocardiogram
EF: Ejection fraction
LA: Left atrium
LPA: Left pulmonary artery
LV: Left ventricle
PAP: Pulmonary artery pressure
PDA: Patent ductus arteriosus
PH: Pulmonary hypertension
PVR: Pulmonary vascular resistance
SVT: Supraventricular tachycardia
TV: Tricuspid valve
VSD: Ventricular septal defect
WU: Woods unit
